# Healthcare use according to deprivation among French Alzheimer's Disease and Related Diseases subjects: a national cross-sectional descriptive study based on the FRA-DEM cohort

**DOI:** 10.3389/fpubh.2024.1284542

**Published:** 2024-02-29

**Authors:** Anaïs Couret, Maryse Lapeyre-Mestre, Axel Renoux, Virginie Gardette

**Affiliations:** ^1^Agence Régionale de Santé Occitanie, Toulouse, France; ^2^Maintain Aging Research Team, CERPOP, Université de Toulouse, Inserm, Université Paul Sabatier, Toulouse, France; ^3^Centre Hospitalier Universitaire de Toulouse, Department of Pharmacology, Toulouse, France; ^4^Centre d'Investigation Clinique 1436, Team PEPSS “Pharmacologie En Population cohorteS et biobanqueS,” Centre Hospitalier Universitaire de Toulouse, Inserm, Université Paul Sabatier, Toulouse, France; ^5^Centre Hospitalier Universitaire de Toulouse, Toulouse, France; ^6^Centre Hospitalier Universitaire de Toulouse, Department of Epidemiology and Public Health, Toulouse, France

**Keywords:** dementia – Alzheimer's disease, deprivation, health resource use, social inequalities, FRA-DEM cohort

## Abstract

**Introduction:**

Pluriprofessional and coordinated healthcare use is recommended for Alzheimer's Disease and Related Diseases (ADRD). Despite a protective health system, France is characterized by persistent and significant social inequalities in health. Although social health inequalities are well documented, less is known about social disparities in healthcare use in ADRD, especially in France. Therefore, this study aimed to describe healthcare use according to socioeconomic deprivation among ADRD subjects and the possible potentiating role of deprivation by age.

**Methods:**

We studied subjects identified with incident ADRD in 2017 in the French health insurance database (SNDS). We described a large extent of their healthcare use during the year following their ADRD identification. Deprivation was assessed through French deprivation index (Fdep), measured at the municipality level, and categorized into quintiles. We compared healthcare use according to the Fdep quintiles through chi-square tests. We stratified the description of certain healthcare uses by age groups (40–64 years, 65–74 years, 75–84 years, 85 years, and older), number of comorbidities (0, 1, 2–3, 4 comorbidities and more), or the presence of psychiatric comorbidity.

**Results:**

In total, 124,441 subjects were included. The most deprived subjects had less use of physiotherapy (28.56% vs. 38.24%), ambulatory specialists (27.24% vs. 34.07%), ambulatory speech therapy (6.35% vs. 16.64%), preventive consultations (62.34% vs. 69.65%), and were less institutionalized (28.09% vs. 31.33%) than the less deprived ones. Conversely, they were more exposed to antipsychotics (11.16% vs. 8.43%), benzodiazepines (24.34% vs. 19.07%), hospital emergency care (63.84% vs. 57.57%), and potentially avoidable hospitalizations (12.04% vs. 10.95%) than the less deprived ones.

**Discussion and conclusion:**

The healthcare use of subjects with ADRD in France differed according to the deprivation index, suggesting potential health renunciation as in other diseases. These social inequalities may be driven by financial barriers and lower education levels, which contribute to health literacy (especially for preventive care). Further studies may explore them.

## Introduction

Health inequities are defined as “systematic differences in the health status of different population groups” (1). Among them, socioeconomic inequalities refer to the differential exposure to risk factors for health (2), along with the utilization of health services and unmet healthcare needs (3, 4). For example, for a comparable level of need, poorer individuals are less likely to see a doctor than wealthier ones, with this difference being more marked for specialists (3, 5).

Deprivation refers to a state of “disadvantage relative to the local community […] to which an individual, family or group belongs,” as defined by Townsend (6). In epidemiology, various deprivation indices have been developed at a geographic area level (6–9). The effect of deprivation on health has been widely studied (9–12), and deprivation can constitute a barrier to accessing healthcare (11). These effects are also seen in Alzheimer's Disease and Related Diseases (ADRD), an inexorable disease that affects over 50 million people worldwide (13). Facing the diversity of ADRD symptoms, including cognitive, functional, and sometimes behavioral and psychological symptoms of dementia (BPSD), a pluriprofessional and coordinated approach to care is required (14, 15). In ADRD, deprivation has been suggested to be associated with a delayed diagnosis (16–18) and a stronger mortality (19, 20). It could complicate access to such recommended healthcare or be associated with non-recommended healthcare use (18, 21, 22), such as emergency care or inappropriate psychotropic drug use. However, there is scarce literature about global healthcare use (not focusing on only a part of healthcare) among subjects with ADRD according to deprivation, especially recommended healthcare (such as physiotherapy or nursing care).

France is recognized for its particular attention to social protection and healthcare access equality, with an extensive healthcare system as compared to other countries (23). It offers healthcare reimbursement to any stable and regular resident or worker in France through the mandatory public part of the French insurance health system (“*Assurance Maladie”*). Most healthcare is predominantly reimbursed by the “*Assurance Maladie*” (i.e., 70% for a medical visit or 65% for medically important drugs), with the remaining part corresponding to out-of-pocket expenses. The latter can be minimized through complementary health insurance and full exoneration of healthcare for some costly chronic diseases, such as ADRD. However, there is still a variable out-of-pocket expense (21, 22). Despite its high level of investment in healthcare provision and social protection, France is characterized by wider social inequalities in mortality (24) and persistent significant social health inequalities (25), leading to the implementation of policies to reduce social health inequities (23, 26, 27). Although social inequalities in health are documented, less is known about social disparities in healthcare use among French subjects with ADRD.

Therefore, this study aimed to describe a wide range of healthcare use according to deprivation among French subjects with ADRD, with particular attention on recommended healthcare, such as physiotherapy or nursing care.

## Materials and methods

### Data source

This study was conducted in a population-based prospective cohort (FRA-DEM), which is an open dynamic cohort, gathering all presumed incident subjects with ADRD identified since 2011 in the French health insurance database [Système National des Données de Santé (SNDS)] (28, 29). SNDS includes all reimbursed healthcare use (ambulatory and hospital care, drug reimbursements). SNDS also gathers diagnoses and medical history during hospital stays and medically confirmed chronic diseases recorded in a long-term diseases (LTD) registry. LTD allows full coverage for related healthcare uses. The Resid-EHPAD database (30), which collects nursing home (NH) stays data, was also made available for the FRA-DEM cohort. Subjects with ADRD were identified if they met at least one of the following criteria: (1) at least two reimbursements of anti-dementia drugs (anticholinesterase inhibitors or memantine), (2) hospitalization with ADRD diagnosis (ICD-10 codes: “F00-F03,” “G30,” “G31” except “G31.2” and “G31.8”), and (3) ADRD LTD registration (ICD-10 codes: “F00-F03,” “G30,” “G31” except “G31.2) (31). A subject was considered to have an incident with ADRD if he did not present any of the previous criteria during the 5 years preceding the identification date.

FRA-DEM received approval from the French data protection authority (CNIL authorization n°1631786-DE-2013–037).

### Study population

For the present study, we focused on subjects identified with incident ADRD in 2017 in the FRA-DEM cohort and affiliated with the main scheme, “Régime Général.”

### Deprivation

We explored the subjects' deprivation through the French ecological deprivation index (Fdep) based on the subjects' municipality of residence (9). This indicator was built on several data measured at the municipality level: the unemployment rate in the active population (15–64 years old), labor rate in the active population, rate of high school graduates in the out-of-school population over 15 years old, and reported median income per household consumption unit. Then, it was categorized into quintiles (Q5 being the most deprived) at the French general population level. The subjects' municipality was defined as the most reliable known area of residence 1 year before the ADRD identification date in the SNDS.

We studied healthcare use (ambulatory and hospital settings, drug reimbursement, and institutionalization) during the year following ADRD identification (i.e., until the end of 2018 for subjects identified as incidents at the end of 2017).

### Ambulatory medical consultations

We described the use of ambulatory medical consultations through three variables:

- the number of General Practitioner (GP) consultations (0–1, 2–4, 5–7, more than 7);
- the use of ambulatory neurologist or psychiatrist consultation (none or at least once). Those medical specialties are identified as ADRD specialties. The geriatrics being most of the time employed by public hospitals, their activity was not available in the SNDS. The anti-dementia drugs have to be prescribed for the first time by ADRD specialists in France;
- the number of private specialist consultations (with the same categories used for GP consultations). This variable grouped the use of ambulatory consultations with specialists in cardiology, oncology, endocrinology, internal medicine, pulmonology, gastroenterology, hepatology, nephrology, hematology, surgery, ear/nose/throat specialty, dermatology, or rheumatology.

### Ambulatory non-medical consultations

We also considered the use of ambulatory non-medical consultations through:

- the number of ambulatory nursing acts of hygiene care (none, between 1 and 4, or 5 and more), defined as acts of patient hygiene care and monitoring concerning medication intake, for example;
- the number of ambulatory nursing technical acts (same categorization as nursing acts of hygiene care), defined as medical technical acts such as injection, chemotherapy, and wound dressings;
- the use of ambulatory speech therapy sessions (none or at least once).

### Drug reimbursement

We studied drug exposure through drug reimbursement during the year. Various variables were considered:

- the number of reimbursed drugs, categorized as “no drug reimbursement during the year,” “between 1 and 9 reimbursed drugs during a quarter,” or “excessive polypharmacy (quarter).” We used a consensual definition for excessive polypharmacy (32), as defined by more than 10 different substances (a unique ATC code) reimbursed during a quarter. We kept the highest value obtained during the year;
- the number of annual anti-dementia drug reimbursements (none, once, or at least twice);
- the number of annual antipsychotic reimbursements (none, once, or at least twice);
- the number of annual benzodiazepine reimbursements (none, once or twice, or at least three times).

### Hospital care

We studied the use of hospital care through:

- the number of emergency room visits (not followed by a hospitalization), categorized as “none,” “once,” and “at least twice”;
- the use of unplanned hospitalization, including all hospitalizations with an entry via the emergency room (none or at least once);
- the use of potentially avoidable hospitalization (none or at least once), as defined by the Agency for Healthcare Research and Quality (33, 34).

### Preventive care

We studied the use of preventive care during the year through:

- ambulatory preventive consultation (none or at least once), which was defined as the use of ambulatory dentist, gynecologist, midwife, or ophthalmologist consultations;
- vaccination (none or at least once), defined as the reimbursement of vaccines against flu, poliomyelitis, tetanus, diphtheria, or pneumococcus.

### Statistical analyses

We graphically presented the 1-year healthcare use of subjects according to their deprivation index. We performed bivariate tests (chi-square tests) to assess differences by deprivation index quintiles. Considering the multiple tests performed, we applied the Bonferroni correction to avoid α-risk inflation. Therefore, the significance threshold was established at a *p*-value of 10^−3^. The original threshold was established at a *p*-value of 0.05, and we performed tests on more than 40 healthcare use variables (all results not shown).

Due to financial circuits in hospitals and some nursing homes, drug reimbursements cannot be assessed during hospitalizations or institutionalization in a nursing home with an internal pharmacy. Moreover, nursing care cannot be measured for institutionalized or hospitalized subjects. Thus, we described these healthcare uses for community-dwelling subjects only, defined as subjects living at home or institutionalized or hospitalized for a maximum duration of three months during the year. Similarly, physiotherapy sessions could not be measured in NH with global payment. Thus, we stratified the description of physiotherapy sessions by place of life: community-dwelling subjects (as defined above) and institutionalization during more than 3 months of the year.

For some healthcare uses (institutionalization, consultation with a private neurologist or psychiatrist, and physiotherapy sessions), we stratified the analyses by age groups to better apprehend the differences in the effect of deprivation on healthcare uses across age groups (40–65 years, 65–74 years, 75–84 years, 85 years, and older). We also stratified the description of nursing care by the number of comorbidities as of 31 December 2017 (0, 1, 2–3, 4 comorbidities, and more) obtained through a comorbidity mapping developed from the healthcare reimbursement in the SNDS (35). Finally, we stratified the description of antipsychotic and benzodiazepine reimbursements based on the presence of psychiatric comorbidity (according to the comorbidities mapping).

## Results

A total of 124,441 subjects were included. Their sociodemographic characteristics and number of comorbidities according to their deprivation index are presented in Table 1. The most deprived subjects were younger than the less deprived ones: 48.52% of the subjects belonging to Q5 of the Fdep were 85 years and older vs. 54.25% of Fdep Q1 subjects. The most deprived subjects lived more frequently in a rural municipality than the less deprived ones (Q5: 19.67% vs. Q1: 7.63%). The psychiatric comorbidities according to the deprivation index are presented in Supplementary Table 1. Overall, the most deprived subjects had more psychiatric disorders (especially addictive disorders), except for the neurotic mood disorder, which included depressive disorders and other psychiatric disorders.

**Table 1:** Description of the population study's sociodemographic characteristics according to the deprivation index Fdep (*n* = 124,441).

	**Deprivation index Fdep** ^*^												*p*-value^**^
	**Q1**		**Q2**		**Q3**		**Q4**		**Q5**		**Total**		
**Sex**													
Men	8,604	(35.74%)	8,575	(35.45%)	8,793	(35.33%)	9,099	(35.59%)	9,225	(35.86%)	44,296	(35.60%)	0.738
Women	15,468	(64.26%)	18,613	(64.55%)	16,096	(64.67%)	16,466	(64.41%)	16,502	(64.14%)	80,145	(64.40%)	
**Age (at the end of the index year), mean, SD**	83.94	(8.93)	83.32	(9.10)	83.03	(9.19)	82.87	(9.11)	82.73	(9.15)	83.16	(9.11)	< 0.001
**Age groups**													
< 65 years	786	(3.27%)	988	(4.08%)	1,129	(4.54%)	1,078	(4.22%)	1,156	(4.49%)	5,137	(4.13%)	< 0.001
65–74 years	2,333	(9.69%)	2,507	(10.36%)	2,751	(11.05%)	2,912	(11.39%)	3,011	(11.70%)	13,514	(10.86%)	
75–84 years	7,893	(32.79%)	8,147	(33.68%)	8,536	(34.30%)	8,899	(34.81%)	9,078	(35.29%)	42,553	(34.20%)	
85 years and older	13,06	(54.25%)	12,546	(51.88%)	12,473	(50.11%)	12,917	(49.58%)	12,482	(48.52%)	63,237	(50.81%)	
**Type of municipality of residence**													
Urban municipality	22,234	(92.36%)	20,394	(84.31%)	20,140	(80.92%)	19,394	(75.86%)	20,588	(80.02%)	102,750	(82.57%)	< 0.001
Rural municipality	1,837	(7.63%)	3,454	(14.28%)	4,737	(19.03%)	6,166	(24.12%)	5,06	(19.67%)	21,254	(17.08%)	
Missing	1	(0.01%)	340	(1.41%)	12	(0.05%)	5	(0.02%)	79	(0.31%)	437	(0.35%)	
**Number of comorbidities**													
0 or 1 comorbidity	1,456	(6.05%)	1,656	(6.85%)	1,622	(6.52%)	1,649	(6.45%)	1,58	(6.14%)	7,963	(6.40%)	< 0.001
2 or 3 comorbidities	5,489	(22.80%)	5,506	(22.76%)	5,674	(22.80%)	5,691	(22.26%)	5,596	(21.75%)	27,956	(22.47%)	
4 comorbidities and more	17,127	(71.15%)	17,026	(70.39%)	17,593	(70.69%)	18,225	(71.29%)	18,551	(72.11%)	88,522	(71.13%)	
**Psychiatric comorbidity**													
No	16,722	(69.47%)	17,135	(70.84%)	17,355	(69.73%)	17,900	(70.02%)	18,261	(70.98%)	87.373	(70.21%)	< 0.001
Yes	7,350	(30.53%)	7,053	(29.16%)	7,534	(30.27%)	7,655	(29.98%)	7,466	(29.02%)	37.068	(29.79%)	

^*^From Q1 the less deprived to Q5 the most deprived.^**^Kruskal–Wallis equality-of-populations rank test for age at the end of the index year and chi^2^ tests for categorical variables.

### ADRD LTD

Overall, during the year following the ADRD identification, an ADRD LTD was slightly more frequently recorded among the less deprived subjects (37.40% among Fdep Q1, 36.09% in Fdep Q5) (*p* < 0.001).

### Anti-dementia drug reimbursement

There was no significant difference in the anti-dementia drug reimbursement according to the Fdep after the Bonferroni correction (14.73% of Fdep Q5 community-dwelling subjects had at least two anti-dementia drug reimbursements vs. 15.14% of Fdep Q1 community-dwelling subjects).

### Institutionalization

The most deprived subjects were less institutionalized than the less deprived ones (Q5: 28.09% vs. Q1: 31.33%) (Supplementary Figure 1).

### Ambulatory medical consultations

**Figure 1:**
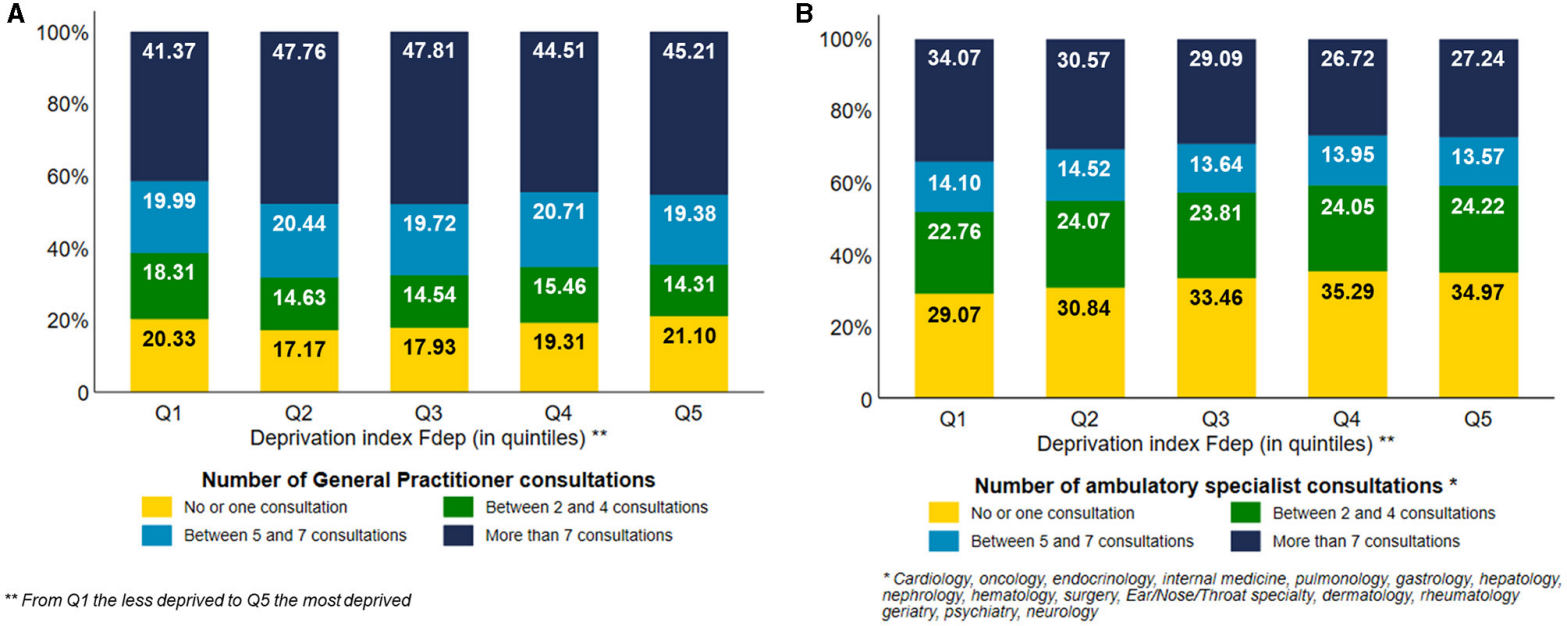
Distribution of the number of general practitioner consultations **(A)** and the number of ambulatory specialist consultations **(B)** according to the deprivation index Fdep (*n* = 124,441).

**Figure 2:**
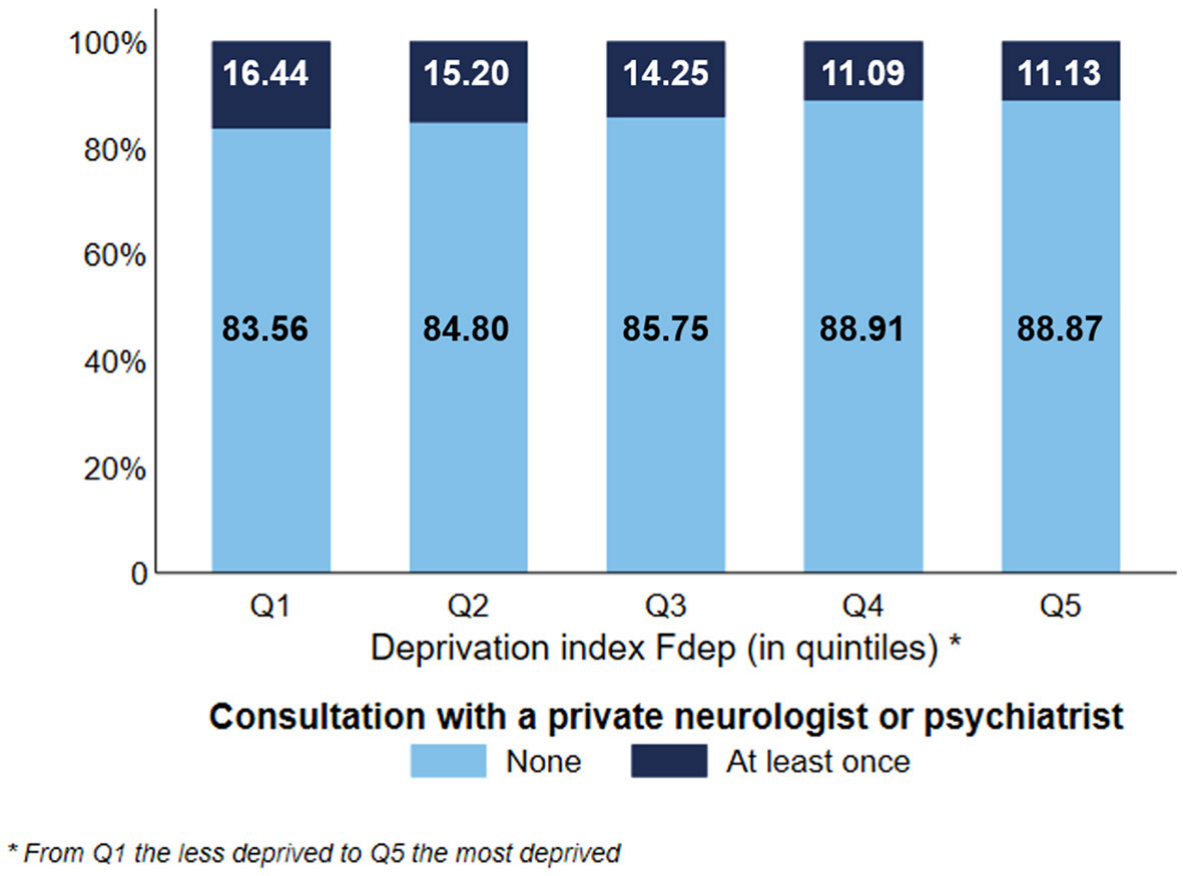
Distribution of the use of private neurologist or psychiatrist consultation according to the deprivation index Fdep (*n* = 124,441).

Concerning the use of GP consultations (Figure 1A), we observed a gradient effect of the deprivation index, except for subjects belonging to the Fdep Q1. 45.21% of the most deprived subjects used more than seven consultations vs. 47.76% of the subjects belonging to Fdep 2. Moreover, the most deprived subjects used fewer ambulatory specialist consultations (Figure 1B) than the less deprived ones, with a gradient effect (27.24% of Fdep Q5 subjects used more than seven consultations vs. 34.37% of Fdep Q1 subjects). When focusing on the use of private neurologist or psychiatrist consultation (Figure 2), 11.13% of the subjects belonging to the Fdep Q5 used at least one consultation vs. 16.44% of the Fdep Q1 subjects, with a visual gradient effect. This difference was particularly marked in the 85 years and older group (Q5: 5.86% vs. Q1: 10.44%) (Supplementary Figure 2).

### Ambulatory non-medical consultations

**Figure 3:**
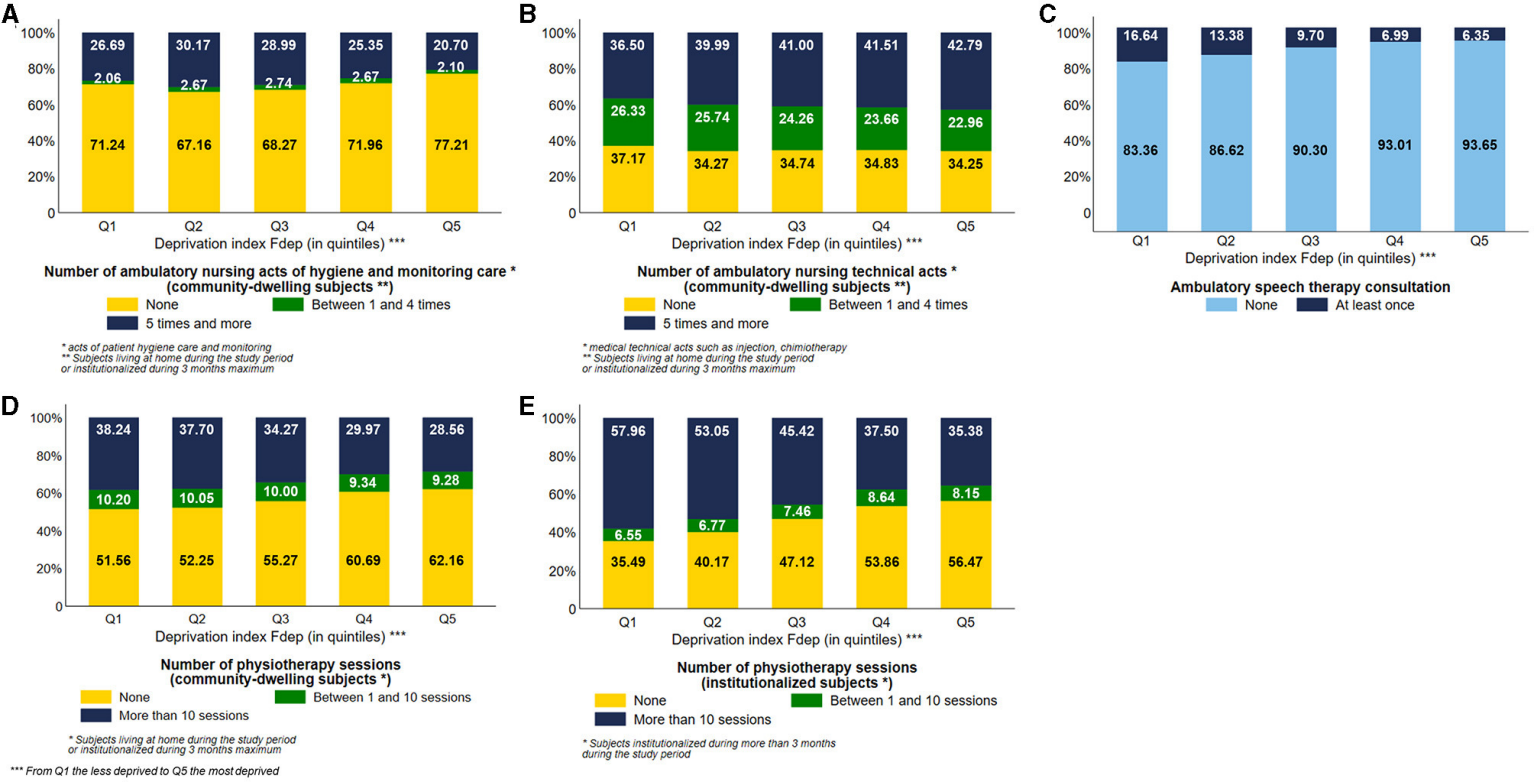
Distribution of the number of ambulatory nursing acts of hygiene care among community-dwelling subjects **(A)**, number of ambulatory nursing technical acts among community-dwelling subjects **(B)**, use of ambulatory speech therapy consultation **(C)**, number of physiotherapy sessions among community-dwelling subjects **(D)**, and number of physiotherapy sessions among institutionalized subjects **(E)** according to the deprivation index Fdep (*n* = 95,653 community-dwelling subjects and *n* = 28,788 institutionalized subjects; *n* = 124,441 subjects in total).

Considering the use of ambulatory nursing acts by community-dwelling subjects, the most deprived subjects used ambulatory nursing acts of hygiene and monitoring care less often than the less deprived ones. 20.70% of Fdep Q5 subjects used at least five times this type of nursing care vs. 26.69% of Fdep Q1 subjects (Figure 3A). On the contrary, 42.79% of the most deprived subjects used ambulatory nursing technical acts at least five times vs. 36.50% of the less deprived subjects, with a visual gradient effect of the Fdep on this healthcare use (Figure 3B). We stratified those analyses based on the number of comorbidities (0, 1, 2 or 3, 4, and more) (Supplementary Figure 3). Regardless of the number of comorbidities, we observed the same differences in the use of ambulatory nursing acts according to the deprivation index.

Concerning speech therapy (Figure 3C), the most deprived subjects used fewer ambulatory speech therapy sessions than the less deprived subjects, with a visual gradient effect (Q5: 6.35% vs. Q1: 16.64%).

The most deprived subjects, institutionalized or not, used fewer physiotherapy sessions than the less deprived ones, with a visual gradient effect of the Fdep on this healthcare use (28.56% of Fdep Q5 community-dwelling subjects used more than 10 sessions vs. 38.24% of community-dwelling subjects belonging to the Fdep Q1) (Figures 3D, E). This difference was particularly prevalent in the 85 years and older group, with 28.20% of community-dwelling subjects belonging to the Fdep Q5 using more than 10 physiotherapy sessions vs. 40.46% of Fdep Q1 community-dwelling subjects (Supplementary Figure 4).

### Drug reimbursement

**Figure 4:**
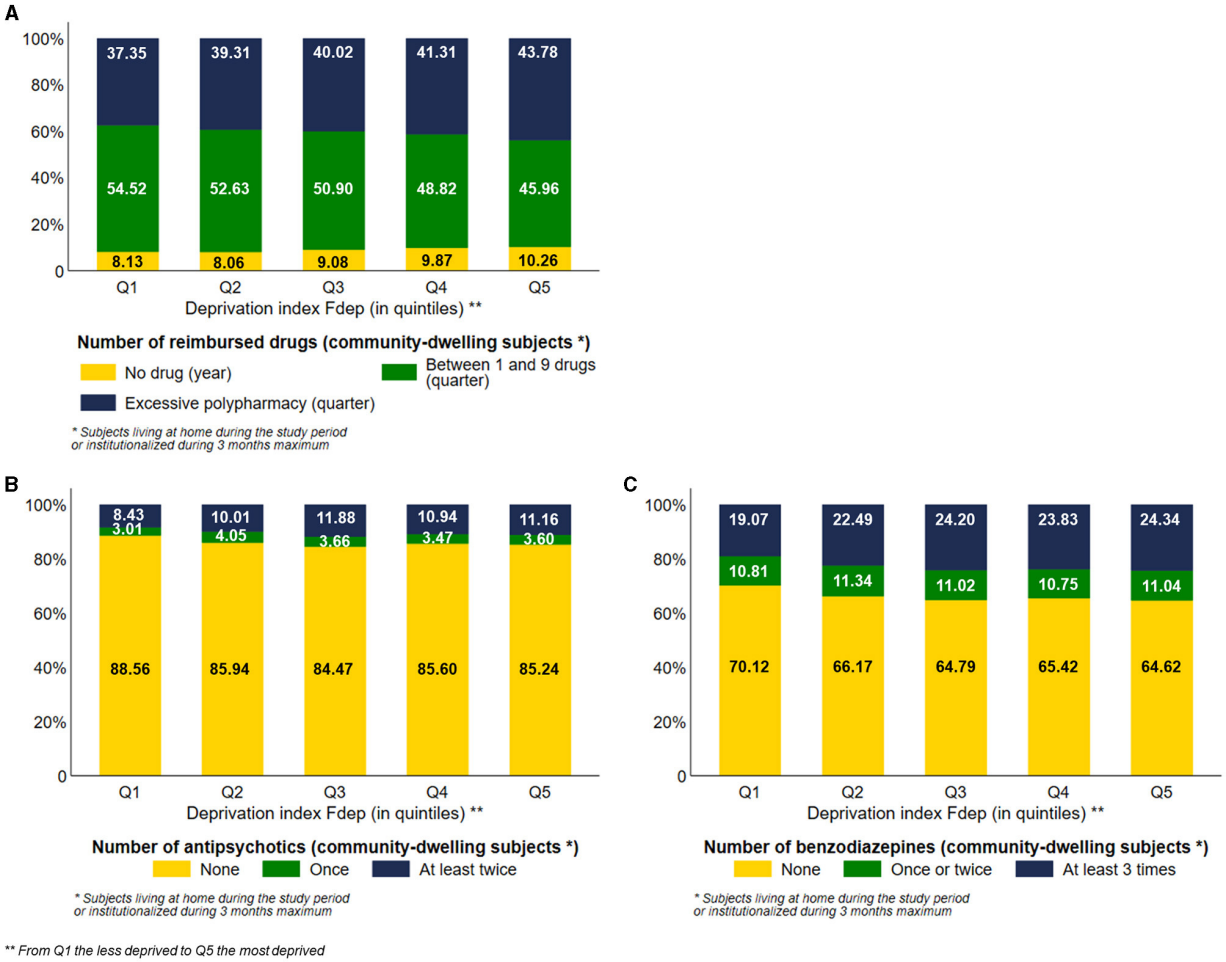
Distribution of the number of reimbursed drugs **(A)**, the number of antipsychotic medication dispensed **(B)**, and the number of benzodiazepine medication dispensed **(C)** among community-dwelling subjects according to the deprivation index Fdep (*n* = 95,653 community-dwelling subjects).

Among community-dwelling subjects, 43.78% of the most deprived subjects were exposed to excessive polypharmacy vs. 37.35% of the less deprived ones, with a visual gradient effect of the Fdep on this aspect of healthcare use (Figure 4A). Among community-dwelling subjects, 10.26% of Fdep Q5 subjects did not have any drug reimbursement during the year vs. 8.13% of the subjects belonging to Fdep Q1, with a gradient effect (Figure 4A). We stratified those analyses based on the number of comorbidities (0, 1, 2 or 3, 4, and more) (Supplementary Figure 5). Regardless of the number of comorbidities, we observed the same differences in the number of reimbursed drugs according to the deprivation index.

Among community-dwelling subjects, the most deprived subjects were more exposed to antipsychotics and benzodiazepines than the less deprived ones, with a visual gradient effect of the Fdep for both healthcare uses: 11.16% and 24.34% of Fdep Q5 subjects had at least two reimbursements of antipsychotics and benzodiazepines, respectively, vs. 8.43% and 19.07% of subjects belonging to Fdep Q1, respectively (Figures 4B, C). We stratified those analyses based on the presence of psychiatric comorbidities (Supplementary Figure 6). There was a greater difference in antipsychotics and benzodiazepines exposure according to the deprivation index in the presence of psychiatric comorbidity.

### Hospital care

**Figure 5:**
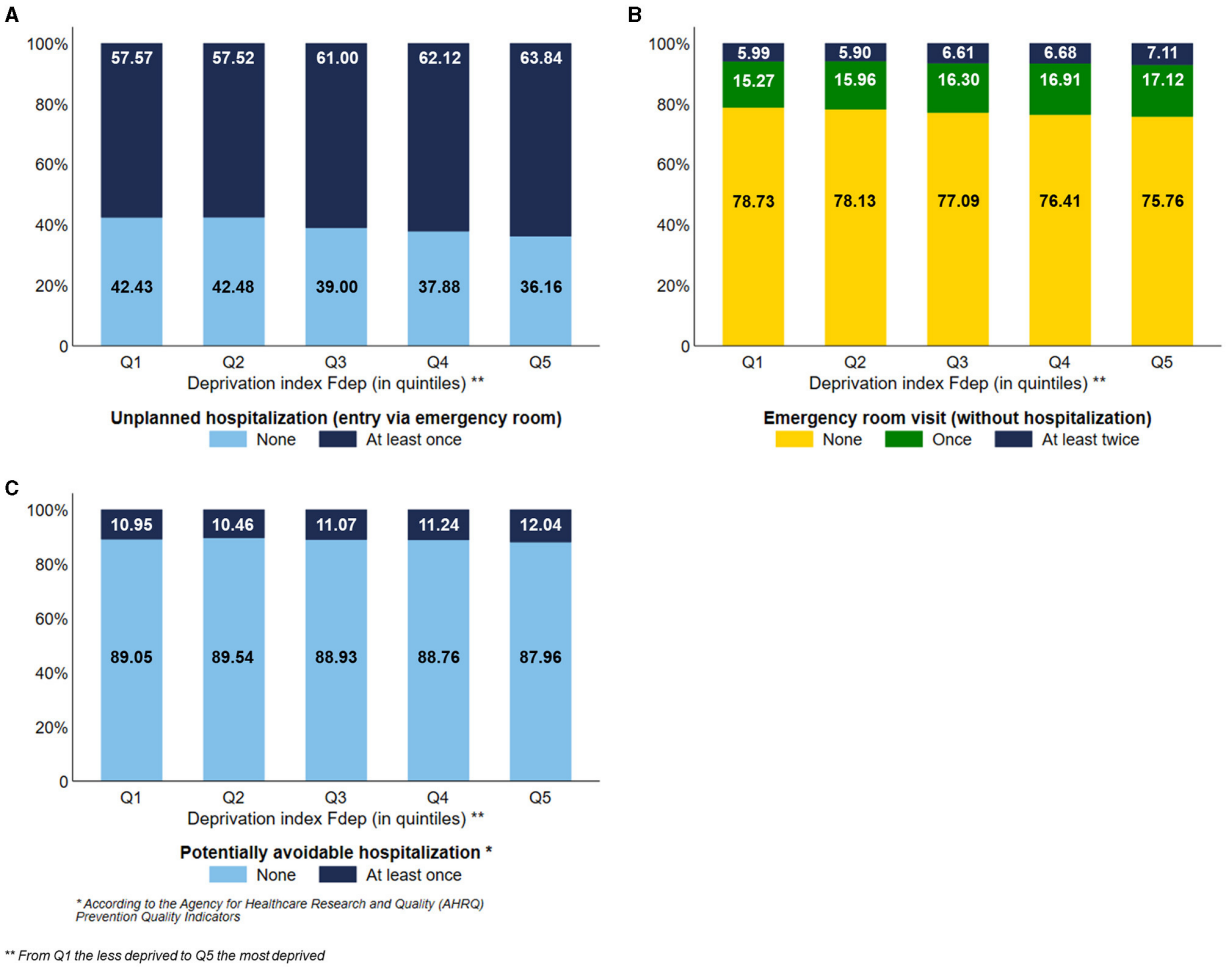
Distribution of the use of unplanned hospitalization **(A)**, emergency room visits **(B)**, and potentially avoidable hospitalization **(C)** according to the deprivation index Fdep (*n* = 124,441).

The most deprived subjects used more unplanned hospitalization than the less deprived ones, with a visual gradient effect of the Fdep (Figure 5A) (Q5: 63.84% vs. Q1: 57.57%). To a lesser extent, the most deprived subjects used more emergency room visits without hospitalization than the less deprived ones, with a gradient effect of the Fdep (Figure 5B). Subjects belonging to the Fdep Q5 were more exposed to potentially avoidable hospitalization than the Fdep Q1 subjects, 12.04% and 10.95%, respectively, with a visual gradient effect of the Fdep (Figure 5C).

### Preventive care

**Figure 6:**
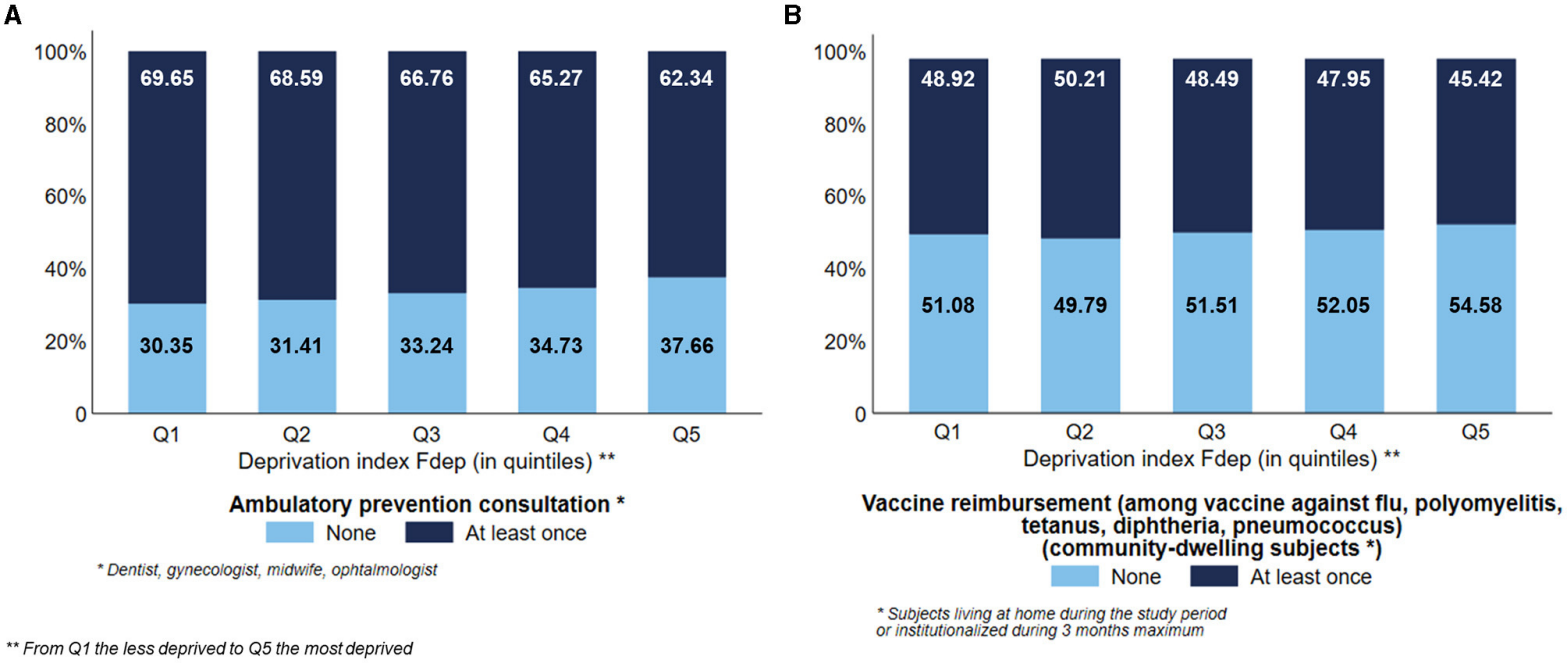
Distribution of the use of ambulatory preventive consultation (*n* = 124,441) **(A)** and vaccine reimbursement among community-dwelling subjects (*n* = 95,653) **(B)** according to the deprivation index Fdep.

The most deprived subjects used less preventive care than the least deprived ones, with a visual gradient effect of the Fdep: 62.34% and 46.67% of the subjects belonging to the Fdep Q5 used ambulatory preventive consultation and vaccination, respectively, vs. 69.65% and 49.45% of the subjects belonging to the Fdep Q1, respectively (Figures 6A, B).

## Discussion

This descriptive study underlines differences in healthcare use among French ADRD subjects according to their deprivation index. Overall, there was less recommended healthcare use among the most deprived subjects, whether the healthcare use was ADRD-related or not. These differences in healthcare use were sometimes amplified by age, with greater differences in older subjects.

In France, a part of healthcare use is reimbursed by the “Assurance Maladie”. Subjects can subscribe to complementary health insurance to be reimbursed for the copayment (with variable reimbursement rates, according to the subscribed contract). However, an incompressible and capped out-of-pocket remains to make the system users accountable (medical franchise, hospital charge, and “participation forfaitaire”). A medical franchise of 0.5€ is applied for each drug box dispensed and for each paramedical act (including nursing care, physiotherapy, and speech therapy sessions), leading to a deduction from reimbursement of 50€ per year. The hospital charge consists of the cost of accommodation and maintenance when the duration of hospitalization exceeds 1 day. The “participation forfaitaire” refers to a franchise of 1€ applied for each consultation or act with a GP or specialist in radiology and biology procedures, leading to a deduction from reimbursement in a limit of 50€ per year. In total, 95% of the French population have subscribed to complementary health insurance (23, 36). As the subscription price to complementary health insurance usually increases with age (considering an increased risk of health expenses with age), older people are one of the most uncovered subgroups, despite financial aid. If they benefit from complementary health insurance, they may have less generous contracts (23). For the most indigent subjects, this complementary health insurance is almost free and offered by the public system. An LTD registration for some medically confirmed chronic diseases (such as ADRD) allows full reimbursement for related healthcare uses. However, the patients are not exempted from the medical franchise, the overbilling (occurring in the case of healthcare use in the private sector), the hospital charge, and the “participation forfaitaire”. Therefore, even with an LTD registration, the financial barrier may persist for some types of healthcare (23, 36, 37). In 2016, the patients with an LTD registration had, on average, an out-of-pocket of 820€ per year (38).

Therefore, some differences in healthcare use according to the deprivation index could be explained by economic barriers. ADRD-unrelated healthcare uses such as ambulatory specialists, preventive consultations, and vaccination were less frequent among the most deprived subjects. However, regardless of the deprivation index, the low vaccination rate should be noted in this fragile population (<50%, even though vaccination is recommended and fully reimbursed for this population) (39, 40). Then, the most deprived subjects had more frequently no reimbursed drug during the year. Moreover, ADRD-related healthcare uses were also less frequent among the most deprived subjects (private neurologist or psychiatrist consultation, speech therapy, and physiotherapy sessions). These healthcare uses are concerned with the previously described medical franchise, or ‘participation forfaitaire', to which must be added the copayment (which represents 40% of the cost of physiotherapy sessions, for example) or the cost of private insurance for the copayment reimbursement (with variable reimbursement rates). In 2017, ~45% of the ambulatory specialists were eligible to perceive overbillings, varying between 10% and 115% higher than the conventional tariffs, depending on the geographical area (23). Furthermore, institutionalization occurred less often among the most deprived subjects, which is in line with a Welsh study (41). In 2017, the median price of a single bedroom in a French NH was 1,953€ per month, representing 112% of the average income of a retiree (42). All these differences in healthcare uses according to deprivation could underline the first reason for forgoing healthcare in France, which is the cost represented by the subjects (36). Approximately 20% of French people forego healthcare (43). According to the OECD, the unmet need for medical examinations due to financial reasons is higher for persons with a low income in France than the European Union average (4).

The lower use of recommended healthcare for the most deprived subjects could be explained by psychosocial factors. As explained above, the French health system might be difficult to understand, with complicated procedures for accessing social rights (23) or difficulties in contacting an ambulatory specialist directly for the most deprived subjects. Moreover, the most deprived subjects may have low health education and literacy, with little attention given to their health in comparison with their other needs (such as living conditions). This low health education could lead to poorer compliance with recommended ambulatory and preventive healthcare for the most deprived subjects, leading to greater use of emergency room visits with or without hospitalization (21, 22, 44) and potentially avoidable hospitalization. This poor compliance could also partly explain the greater proportion of most deprived subjects without drug reimbursement during the year. Low health literacy could also lead to greater fatalism among the most deprived subjects and their relatives, who might not see the point of using functional and non-vital healthcare, such as physiotherapy and speech therapy sessions.

Furthermore, in our population, the most deprived subjects were more likely to live in rural municipalities and therefore may encounter more difficulties in accessing healthcare, given that rural areas usually have lower GP and specialists' densities. This geographic isolation could complicate access to ambulatory medical and non-medical healthcare, particularly for the oldest ADRD subjects, who might depend on a third party to take them there. This suboptimal ambulatory follow-up could result in emergency care or potentially avoidable hospitalization.

In line with a UK study, the most deprived subjects used GP consultations (44) and ambulatory nursing acts of hygiene and monitoring care more frequently than the less deprived ones, except for the subjects belonging to the Fdep Q1. This gradient effect could be driven by the greater number of comorbidities among the most deprived subjects (72.11% of Fdep Q5 subjects have at least four comorbidities vs. 70.39% of Fdep Q2 subjects), necessitating a more regular medical follow-up. This lower use of GP consultations among the subjects belonging to the Fdep Q1 could be explained by a shift of GP consultations toward specialists ones; this less deprived population has fewer economic barriers to access such specialized care. Moreover, the subjects belonging to the Fdep Q1 could be accompanied by homecare workers, for which an important out-of-pocket expense remains. SNDS data did not allow us to measure such a phenomenon. This population was also more frequently exposed to excessive polypharmacy, possibly highlighting the burden of comorbidities.

The most deprived subjects were more exposed to antipsychotics and benzodiazepines, as observed elsewhere (18, 45). This could be explained by the existence of BPSD, with a delayed ADRD diagnosis, reported as frequent in this population (16–18). Those drugs are not recommended for subjects suffering from ADRD (except for risperidone), and the prescriber could be influenced by the socio-economic status of the patient, in addition to the fact that the relatives of the most deprived subjects could be less vigilant about such not recommended practices. Moreover, this difference was greater in the presence of psychiatric comorbidities.

This differential healthcare use according to deprivation varied according to age, with increasing differences among older subjects. This could be explained by the fact that this is a more isolated population, with a probable higher proportion of widowers than the younger subjects. To overcome this isolation, subjects may need to mobilize significant financial resources to access healthcare, which may be difficult for the most deprived ones. Moreover, the less deprived older subjects could be overselected, being particularly healthy. Paying particular attention to their health compared to the most deprived subjects, healthcare use inequalities could widen between the most and the least deprived subjects.

Some limitations of our study are related to the use of administrative databases. First, the definition of ADRD identification did not rely on clinical data. Second, some information was not available in the SNDS, such as ADRD severity or etiology, the presence of an informal caregiver, the use of home workers, BPSD, educational level, or lifestyle habitus. Then, the deprivation was measured at a contextual level and not at an individual level. Therefore, it is possible that a deprivation quintile was wrongly attributed to a subject living in the concerned municipality. We chose to focus on healthcare use during the year following ADRD identification because it is during this period that the care of the disease with different health and allied health professionals is organized. Finally, this study focused on the difference in healthcare uses according to deprivation without investigating its appropriateness, meaning that the increased healthcare use in the less deprived subjects may reflect overuse.

Nevertheless, our study presents several strengths. First, we used recent data before the reimbursement withdrawal of anti-dementia drugs in France occurred in 2018 and before the COVID-19 pandemic, which might have impacted healthcare uses. Moreover, we used reliable data reflecting real-life consumption of healthcare, especially regarding ambulatory consultations and hospitalizations. Second, our large population study was representative of the French population, with beneficiaries of the general scheme of health insurance, which covers ~70% of the French population. Finally, this study was the first to investigate differences in healthcare use among French subjects with ADRD in various settings according to their deprivation.

## Conclusion

This study confirms social differences in healthcare use among French subjects suffering from ADRD during the year following ADRD identification, in particular in older adults, despite the efforts made to limit healthcare use renunciation to recommended healthcare use among the most deprived. The need for a voluntarist health education and promotion policies toward the most deprived persists, as well as attention to a healthcare supply policy across the country.

## Data availability statement

The original contributions presented in the study are included in the article/Supplementary material, further inquiries can be directed to the corresponding author.

## Ethics statement

The studies involving humans were approved by the Commission Nationale de l'Informatique et des Libertés. The studies were conducted in accordance with the local legislation and institutional requirements. Written informed consent for participation was not required from the participants or the participants' legal guardians/next of kin because written informed consent from the participants was not required to participate in this study in accordance with the French legislation related to the SNDS (pseudonymised data).

## Author contributions

AC: Conceptualization, Formal analysis, Methodology, Visualization, Writing – original draft. ML-M: Conceptualization, Methodology, Supervision, Writing – review & editing. AR: Conceptualization, Methodology, Supervision, Writing – review & editing. VG: Conceptualization, Methodology, Supervision, Writing – review & editing.
