# Supplementary material for: Healthcare use according to deprivation among French Alzheimer's Disease and Related Diseases subjects: a national cross-sectional descriptive study based on the FRA-DEM cohort
Source: Front Public Health. 2024 Feb 29;12:1284542. doi: 10.3389/fpubh.2024.1284542 (PMC10937384; doi:10.3389/fpubh.2024.1284542)
Supplement: Supplementary file 1 [file Table_1.DOCX]

Supplementary table 1: Psychiatric comorbidities according to the deprivation index Fdep

| **Deprivation index Fdep *** | | | | | | | | | | | | | p-value ** |
| --- | --- | --- | --- | --- | --- | --- | --- | --- | --- | --- | --- | --- | --- |
|  | Q1 | | Q2 | | Q3 | | Q4 | | Q5 | | Total | |  |
| **Psychotic disorder** |  |  |  |  |  |  |  |  |  |  |  |  |  |
| No | 23,286 | (96.73%) | 23,406 | (96.77%) | 23,918 | (96.10%) | 24,700 | (96.62%) | 24,842 | (96.56%) | 120,152 | (96.55%) | < 0.001 *** |
| Yes | 786 | (3.27%) | 782 | (3.23%) | 971 | (3.90%) | 865 | (3.38%) | 885 | (3.44%) | 4,289 | (3.45%) |  |
| **Neurotic mood disorder ****** |  |  |  |  |  |  |  |  |  |  |  |  |  |
| No | 19,532 | (81.14%) | 19,843 | (82.04%) | 20,339 | (81.72%) | 20,935 | (81.89%) | 21,344 | (82.96%) | 101,993 | (81.96%) | < 0.001 *** |
| Yes | 4,540 | (18.86%) | 4,345 | (17.96%) | 4,550 | (18.28%) | 4,630 | (18.11%) | 4,383 | (17.04%) | 22,448 | (18.04%) |  |
| **Mental deficiency** |  |  |  |  |  |  |  |  |  |  |  |  |  |
| No | 24,018 | (99.78%) | 24,106 | (99.66%) | 24,744 | (99.42%) | 25,440 | (99.51%) | 25,607 | (99.53%) | 123,915 | (99.58%) | < 0.001 *** |
| Yes | 54 | (0.22%) | 82 | (0.34%) | 145 | (0.58%) | 125 | (0.49%) | 120 | (0.47%) | 526 | (0.42%) |  |
| **Addictive disorder** |  |  |  |  |  |  |  |  |  |  |  |  |  |
| No | 23,317 | (96.86%) | 23,383 | (96.67%) | 23,952 | (96.24%) | 24,574 | (96.12%) | 24,664 | (95.87%) | 119,890 | (96.34%) | < 0.001 *** |
| Yes | 755 | (3.14%) | 805 | (3.33%) | 937 | (3.76%) | 991 | (3.88%) | 1,063 | (4.13%) | 4,551 | (3.66%) |  |
| **Childhood-onset psychiatric disorders** |  |  |  |  |  |  |  |  |  |  |  |  |  |
| No | 24,011 | (99.75%) | 24,144 | (99.82%) | 24,839 | (99.80%) | 25,507 | (99.77%) | 25,680 | (99.82%) | 124,181 | (99.79%) | 0.348 NS |
| Yes | 61 | (0.25%) | 44 | (0.18%) | 50 | (0.20%) | 58 | (0.23%) | 47 | (0.18%) | 260 | (0.21%) |  |
| **Other psychiatric disorders** |  |  |  |  |  |  |  |  |  |  |  |  |  |
| No | 20,967 | (87.10%) | 21,376 | (88.37%) | 21,911 | (88.03%) | 22,543 | (88.18%) | 22,871 | (88.90%) | 109,668 | (88.13%) | < 0.001 *** |
| Yes | 3,105 | (12.90%) | 2,812 | (11.63%) | 2,978 | (11.97%) | 3,022 | (11.82%) | 2,856 | (11.10%) | 14,773 | (11.87%) |  |
| ** From Q1 the less deprived to Q5 the most deprived* | |  |  |  |  |  |  |  |  |  |  |  |  |
| *** chi² tests for categorical variables* |  |  |  |  |  |  |  |  |  |  |  |  |  |
| ***** Including depressive disorders* |  |  |  |  |  |  |  |  |  |  |  |  |  |
