# Supplementary material for: Healthcare use according to deprivation among French Alzheimer's Disease and Related Diseases subjects: a national cross-sectional descriptive study based on the FRA-DEM cohort
Source: Front Public Health. 2024 Feb 29;12:1284542. doi: 10.3389/fpubh.2024.1284542 (PMC10937384; doi:10.3389/fpubh.2024.1284542)
Supplement: Supplementary file 2 [file Image_1.PDF]

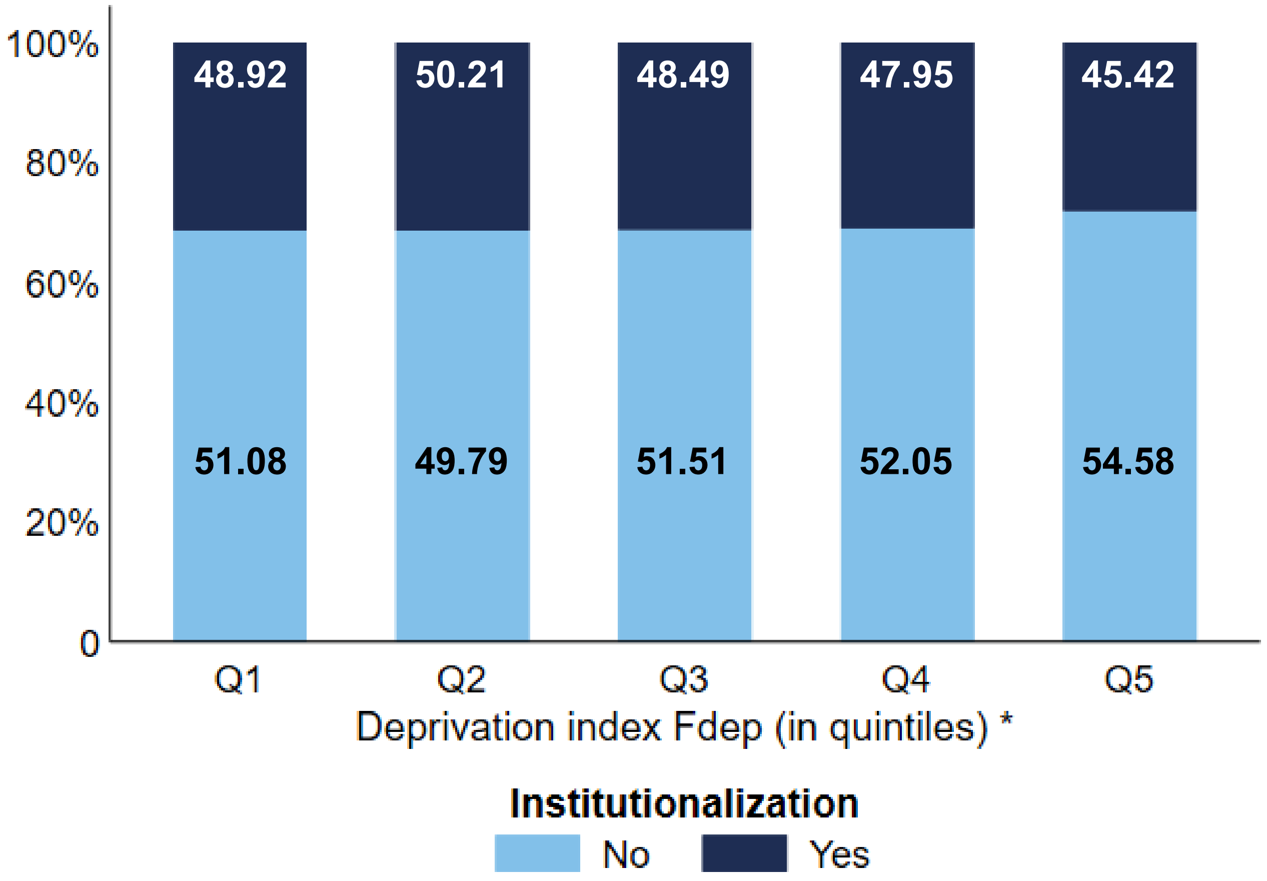

** From Q1 the less deprived to Q5 the most deprived*

Supplementary figure 1: Distribution of the institutionalization according to the deprivation index Fdep (n=124,441)
