# Supplementary material for: Healthcare use according to deprivation among French Alzheimer's Disease and Related Diseases subjects: a national cross-sectional descriptive study based on the FRA-DEM cohort
Source: Front Public Health. 2024 Feb 29;12:1284542. doi: 10.3389/fpubh.2024.1284542 (PMC10937384; doi:10.3389/fpubh.2024.1284542)
Supplement: Supplementary file 7 [file Image_6.PDF]

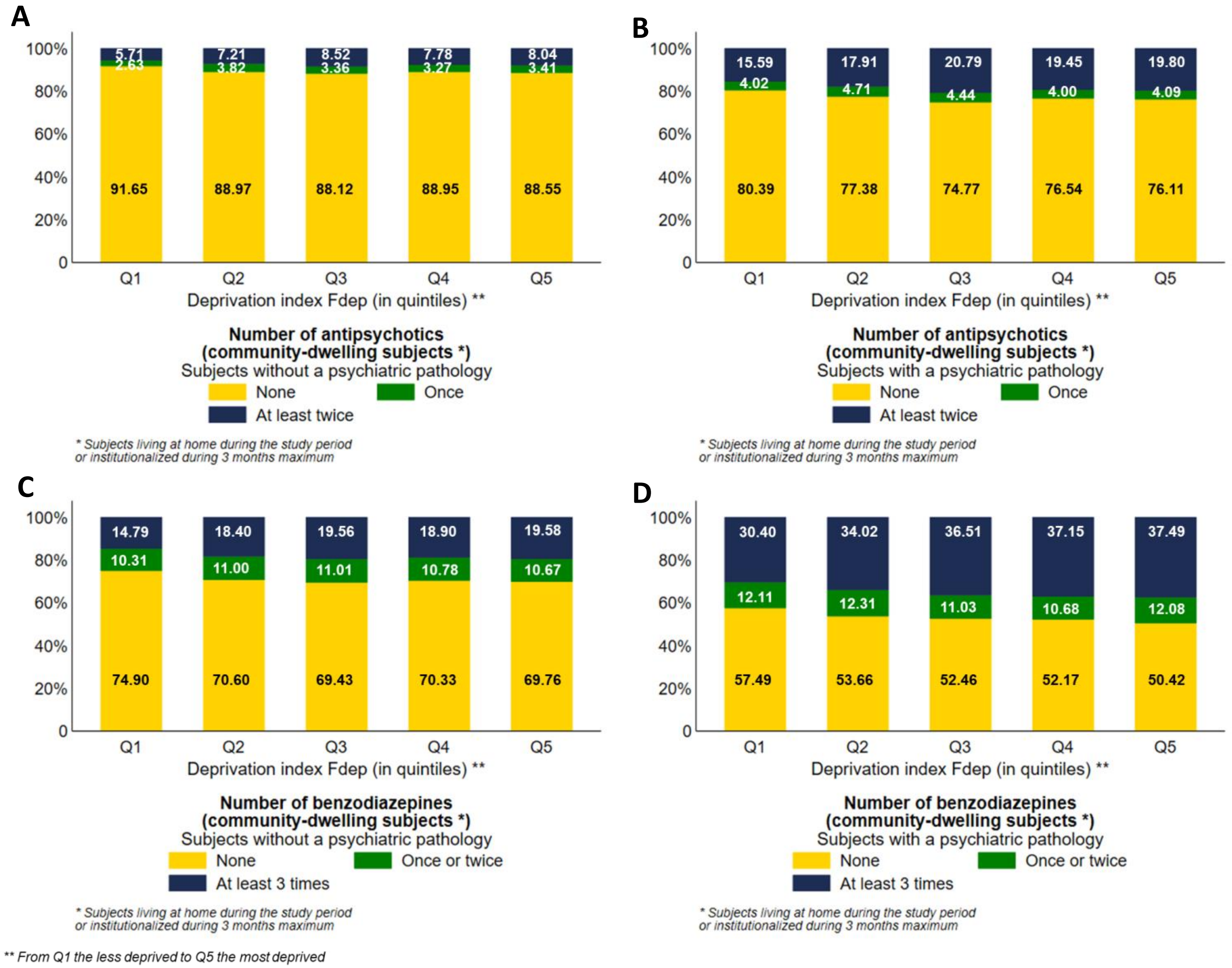

Supplementary figure 6: Distribution of the number of reimbursed antipsychotics (A and B) and benzodiazepines (C and D) according to the deprivation index Fdep, stratified by the presence of psychiatric comorbidity (n=95,653 community-dwelling subjects)
